# Mercury vapour exposure during dental student training in amalgam removal

**DOI:** 10.1186/1745-6673-8-27

**Published:** 2013-10-03

**Authors:** Robin Warwick, Andrea O’Connor, Brianne Lamey

**Affiliations:** 1 Hanna Dental Clinic, 104 Fox Lake Trail, Hanna, Alberta, Canada; 2 House Dental Center, #3, 4910-45 Street, Red Deer, Alberta, Canada; 3 Innisfail Family Dental, 4935 50 St., Innisfail, Alberta, Canada

**Keywords:** Mercury, Mercury vapor, Dental amalgam, Occupational exposure to mercury, Amalgam removal

## Abstract

**Background:**

Amalgam that is used for dental fillings contains approximately 50% elemental mercury. During dental student training, amalgam is often removed by drilling without the use of water spray and suction, which are protective measures in preventing mercury aerosol. In this study we measured mercury vapor levels in ambient air during amalgam removal as is typically performed in dental training.

**Methods:**

Mercury vapor levels in ambient air were measured in a dental school laboratory during removal of amalgam fillings from artificial teeth set into a dental jaw simulator. Mercury vapor was measured under three conditions (25 measurements each): with the simultaneous use of water spray and suction, with the use of suction only, and with the use of neither suction nor water spray. These three conditions are all used during dental student training. Results were compared to Alberta occupational exposure limits for mercury vapor in order to assess potential occupational risk to students. Analysis of variance testing was used to compare data obtained under the three conditions.

**Results:**

When water spray and suction were used, mercury vapor levels ranged from 4.0 to 19.0 μg/m^3^ (arithmetic mean = 8.0 μg/m^3^); when suction only was used, mercury vapor levels ranged from 14.0 to 999.0 (999.0 μg/m^3^ represents the high limit detection of the Jerome analyzer) (arithmetic mean = 141.0 μg/m^3^); when neither suction nor water was used, the vapor levels ranged from 34.0 to 796.0 μg/m^3^ (arithmetic mean = 214.0 μg/m^3^).

**Conclusions:**

The Alberta Occupational Health and Safety threshold limit value for mercury vapor over an eight-hour time-weighted period is 25.0 μg/m^3^. The absolute ceiling for mercury vapor, not to be exceeded at any time, is 125.0 μg/m^3^. When both water spray and suction were used, mercury vapor levels were consistently below this threshold. When suction without water spray was used, mercury vapor levels exceeded the safety threshold 8% of the time. When neither water spray nor suction was used, 36% of the mercury vapor readings exceeded the absolute ceiling value. To maximize safety, dental schools should train students to remove amalgam only while using water spray and high volume suction. Alternatively, students should use appropriate occupational hygiene personal protective equipment during amalgam removals.

## Background

Elemental mercury is a large component (approximately 50%) of dental amalgam. Manipulation of in situ amalgam (as is done during polishing, scaling, and removal with a drill results in vaporization of mercury), results in short-term exposure of mercury vapor to dentists and other dental workers that may exceed occupational safety limits. Dentists are known to have occupational exposure to mercury vapor during these procedures [1]. Many studies have shown that dental workers on average have higher systemic levels of mercury in their tissues and organs than do members of control groups [2-7].

Dental students in Canada and many other countries remove amalgam fillings with dental drills during their training. These procedures are first commonly performed without any measures to reduce or limit mercury exposure; such protective measures include the concomitant use of water spray and/or high-volume suction during drilling. Water spray and suction are generally used in the clinical setting to prevent damage to dental pulp and nerve from heat generated by high-speed drilling. Two common dental procedures in which amalgam may be removed without the protection of water spray and/or high-volume suction are root canal surgery and tooth removal. In these two circumstances, water spray is unnecessary to preserve the pulp because in both cases the pulp is being removed. In other non-clinical settings in which enhanced visibility is needed, dental students are trained to drill on mercury amalgam fillings without protective measures.

Although most government agencies that are responsible for worker safety generally have established thresholds of allowable mercury exposure, it does not ensure that being exposed to levels below these thresholds is safe. The WHO has stated “*studies suggest that mercury may have no threshold below which some adverse effects do not occur”*[8]. Canadian Regulatory bodies involved in worker safety have established safety levels that may not be appropriate for the dental profession [9]. Present thresholds are set predominantly from data involving male chloralkali workers, which is a much different model than the circumstances surrounding mercury exposure in dental clinics. The presence of chlorine in Chloralkali work places protects the worker against mercury exposure. In addition, females and other specific subsets of the population have been identified to have less tolerance to mercury exposure. These factors make it difficult to set a threshold that ensures safety.

The Canada Labour Code and all Provincial Codes have either adopted or specifically referenced the occupational exposure limits for mercury vapor defined as a threshold limit value (TLV) by the American Conference of Governmental Industrial Hygienists (ACGIH) [10-12]. The average TLV for mercury vapor, as prescribed by the ACGIH, is 25.0 μg/m^3^ over an 8-hour period. The ACGIH, Alberta Occupational Health and Safety, and most other provinces also prescribe a maximum ceiling limit of 5 times the TLV (125 μg/m^3^), which is not to be exceeded at any time. Because the study was undertaken in the Province of Alberta, and Alberta Occupational Health and Safety levels are established and enforceable, the data was compared against these limits.

The purpose of this study was to duplicate the conditions of mercury exposure commonly experienced by dental students in Canada performing amalgam removals, to quantify the levels of mercury vapor exposure, and to compare those levels to occupational exposure limits for mercury prescribed in occupational health and safety regulations in Alberta and Canada. We found significantly high levels of mercury vapor in ambient air that exceeded these occupational limits unless both water spray and suction were used.

## Methods

All three authors were dental students at the University of Alberta at the time of the study, which was conducted in a laboratory setting at the dental school. Three researchers (the authors) worked together to collect the data. One researcher performed the amalgam removals, while the second researcher assisted with high- volume suction for water and particulate removal (when required); meanwhile, the third researcher operated a Jerome model 431-X mercury vapor analyzer (Arizona Instrument, LLC, Tempe, AZ) to collect mercury vapor measurements in the ambient air surrounding the dental student performing the removals. This technology was chosen for a number of reasons; the new machine was readily available to the researchers, the Jerome is still relied on in the field by occupational officials to assess conditions where mercury vapor may be an issue, and the Jerome is capable of accurately reading levels in and around the concerned levels set by Alberta Occupational Health and Safety.

**Figure 1:**
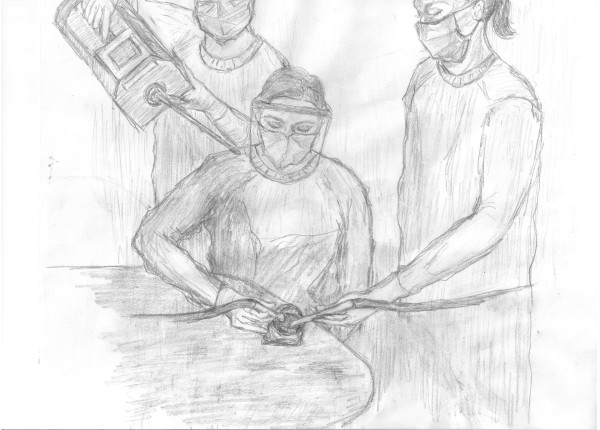
Experimental setup.

All researchers were equipped with Ansell Micro-touch® Powder Free Latex gloves and a disposable ear-loop mask (3 M™ ESPE™ Ear Loop Face Mask, 2000 F). These protective measures are routinely used by students in this laboratory setting. See Figure 1 for a drawing of the experimental setup.

Amalgam removals were performed from upper or lower posterior (molar) teeth on a bench top using amalgam-filled plastic teeth set into a dental jaw simulator. The experimental set-up replicated that of a dental school laboratory. Amalgam removal procedures followed the standard methods prescribed during dental student training at the University of Alberta, which follow accepted standards of dental training in Canada. The removals were performed using a standard high-speed hand piece equipped with a #556 bur. Each filling was prepared as a standard 2-surface molar restoration, involving the occlusal and proximal surfaces, prepared using Dispersalloy® amalgam (50% Mercury, 34.65% Silver, 8.95% Tin, 5.9% Copper, and 0.5% Zinc). These fillings contained an estimated 200 mg to 600 mg of mercury. The amalgam fillings were installed in the teeth of the dental jaw simulator, following accepted techniques, at least one year prior to this study.

A newly purchased, manufacturer-calibrated Jerome 431-X mercury vapor analyzer was used to record air-borne mercury vapor concentrations during the amalgam removal procedures. Regeneration and zeroing of the Jerome analyzer was carried out following the manufacturer’s recommendations [13] throughout the data collection process. The minimum limit of quantification of the Jerome 431-X is 3 μg/m^3^. Care was taken to ensure that no solid or wet materials were drawn into the analyzer during data collection. To mimic the protective equipment worn by a dental student, a disposable ear-loop mask (3 M™ ESPE™ Ear Loop Face Mask, 2000 F) was placed over the intake nozzle of the Jerome analyzer. The mask was replaced after every two mercury vapor concentration recordings. As recommended in the instruction manual, the Jerome analyzer was regenerated on the morning of each data collection day, at the end of each day, and when the Jerome display indicated that regeneration was necessary during the data collection process. Thirty minutes after regeneration, the analyzer was calibrated and then used to collect data. Mercury vapor readings were taken at an average distance of 38 cm from the dental jaw simulator implanted with the amalgam-filled plastic teeth, with the nozzle of the Jerome analyzer pointed directly towards the working area from a superior position, thereby mimicking the position of a dental student’s face during amalgam removal. The distance of 38 cm was determined to be the average distance from the face of the drill operator to the operative site.

When readings were taken with suction alone, and with suction and water spray, one researcher held the suction tube to the left of the researcher performing the removals, with its source coming from the superior position at a 45-degree angle. The tip of the suction device was placed as close to the contact point of the dental bur on the amalgam as was possible.

Amalgam removals were conducted until a total of 75 mercury vapor readings were performed. Twenty-five measurements were conducted under each of these three conditions: with water spray and high-volume suction, with suction only (no water spray), and with neither water spray nor suction. A University of Alberta statistician determined that 25 measurements under each of the three conditions would be sufficient for satisfactory statistical analysis. The time between each vapor measurement varied depending on a number of factors including recalibration and maintenance requirements of the Jerome analyzer, time required to replace amalgam-filled plastic teeth in the dental jaw simulator, and work area clean up.

Non-parametric Mann-Whitney tests were performed on the raw data, and parametric analysis of variance (ANOVA) was performed on the log-transformed mercury concentration data. A University of Alberta statistician performed all of the data analyses.

## Results and discussion

The mean, median, and range of mercury vapor concentrations in ambient air were measured during the removal of dental amalgam fillings with and without water spray and suction (Table 1). The greatest average concentration of mercury vapor was recorded when no suction or water spray was used during amalgam removal. Adding suction lowered average mercury vapor levels; however, the lowest levels of mercury vapor were measured when suction and water spray were both used. In all cases of measurement, mercury vapor concentrations were greater than the limit of detection (> 3 μg/m^3^) of the Jerome analyzer.

**Table 1:** **Measured mercury vapor concentrations (mg/m**^**3**^**) in ambient air during dental amalgam removal**

	**Suction and water spray**	**Suction only (no water spray)**	**No suction and no water spray**
Arithmetic mean and standard deviation (μg/m^3^)	8.0 ± 3.7	142.0 ± 234.6	214 ± 226.4
Median (μg/m^3^)	8.0	68.0^1^	117.0^2^
Range (μg/m^3^)	4.0–19.0	14.0–999.0	34.0–796.0

^1^ Statistically significantly greater (p < 0.001) from Suction & Water.

^2^ Statistically significantly greater (p = 0.031) from Suction & Water.

Table 1 Sample size = 25 for each of the three experimental conditions. During removal of dental amalgams from model teeth in the dental jaw simulator using a dental drill, mercury vapor in ambient air was measured at a distance of 38 cm from the amalgam site.

**Figure 2:**
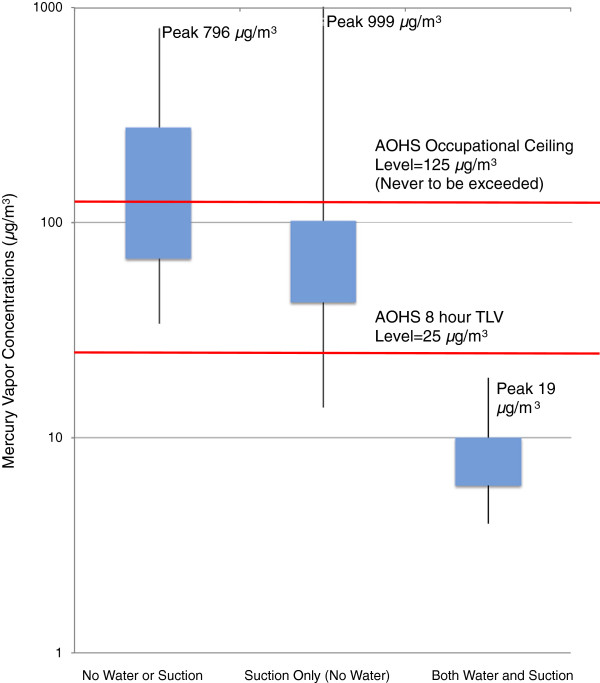
**Mercury vapor concentrations in ambient air during amalgam removal relative to AOHS permissible exposure limits.** Sample size = 25 for each of the three experimental conditions. During removal of dental amalgams from model teeth in the dental jaw simulator using a dental drill, mercury vapor in ambient air was measured at a distance of 38 cm from the amalgam site. The range of measurements and highest measurement obtained under each of the three experimental conditions is portrayed. The thick band indicates the range between the first and fourth quartiles. Alberta Occupational Health and Safety (AOHS) permissible exposure limits for mercury vapor are indicated.

The results of this study demonstrate that the use of suction and water spray during dental amalgam removal procedures significantly reduces the concentration of mercury vapor in ambient air. When suction and water were used, 100% of measured mercury vapor levels were below both the Alberta occupational ceiling limit (AOCL) of 125.0 μg/m^3^ and the Alberta Occupational Threshold Limit Value (AOTLV) of 25.0 μg/m^3^ over an eight-hour time-weighted period for mercury vapor exposure. When suction only (no water spray) was used, the AOCL was breached 8% of the time, with another 16% of the readings close to this limit. In addition, 84% of these readings exceeded the AOTLV. In the absence of both water spray and suction, 100% of the readings exceeded the eight-hour AOTLV of 25.0 μg/m^3^, and 44% of the readings breached the AOCL of 125.0 μg/m^3^. Figure 2 displays the measured mercury vapor concentrations in relation to occupational exposure limits published by Alberta Occupational Health and Safety (AOHS).

With suction and no water as a level of protection, the value range was dramatic (14- > 999 μg/m^3^). The authors hypothesize a number of reasons why this may have occurred. First, the nature of mercury vapor created from a single source (the operative field) in ambient air behaves in a chaotic way, so by chance and by the currents of the ambient air in the room, these mercury plumes may have in some instances concentrated towards the nozzle of the Jerome, and in other times, may have not been proximate to the intake. As well, there could have been some influence of random, volatilizing particulate that landed near the intake of the Jerome, which provided an extra source of mercury vapor during the removal when suction but no water was used. Further studies on the footprint and behavior of mercury contaminated particulate during removal is necessary to fully understand the impact of this source of mercury vapor.

Occupational safety levels for mercury vapor are set by provincial jurisdictions in Canada and may not represent an absolute level of safety. For example, the current time-weighted AOTLV level of 25.0 μg/m^3^ is only 1.0 μg/m^3^ less than the average level shown to cause mild tremors in workers chronically exposed to mercury vapor (26.0 μg/m^3^) [14].

This study suggests that dental students training in amalgam removal without the use of water spray and suction are frequently exposed to mercury vapor levels that exceed established safety standards. In order to prevent exposure to unsafe levels of mercury vapor, suction and water spray should be used during all amalgam removal procedures conducted by dental students (as well as by dental practitioners). Dental students in university laboratories often remove amalgams from plastic teeth without the use of protective measures such as water spray or suction in order to enhance visibility of the amalgam and drill. In clinical cases in which the vitality of a tooth is a not a concern, dental students may be advised to drill on mercury amalgam without using water spray. Although mercury vapor exposure occurs predominantly by inhalation, Alberta Occupational Health and Safety, the ACGIH and all Canadian occupational health and safety regulations make specific reference to the risk of skin exposure and penetration by mercury vapor. In the course of conducting this study, we noted significant accumulation of amalgam particles on clothing and exposed skin. The authors have observed that fellow dental students routinely expose their arms (roll up their sleeves or remove long sleeve outerwear) when working in the dental school laboratories. In situations where exposures exceed occupational limits, such as those reported here during amalgam removals without water or suction, adequate occupational hygiene protective equipment should be considered for student use. Latex gloves commonly worn by dental students are known to be less protective of mercury exposure than non-latex nitrile gloves [15]. Training in proper mercury hygiene is important because dentists and dental workers are at risk of exposure to mercury, the absorption of which has been confirmed by studies that have demonstrated higher systemic levels of this toxin in this population compared to controls. The long-term effects of chronic mercury exposure among dental workers have been established. In an extensive review of the literature Mutter described adverse health issues related to mercury exposure in dental workers that handle dental amalgams [16]. Neurological symptoms were found to be significantly more prevalent among dental assistants compared to non-exposed assistant nurses. These symptoms include psychosomatic symptoms, problems with memory and concentration, fatigue, and sleep disturbance [17]. Female dental assistants working with amalgams have a lower fecundability (ability to conceive) than controls [18]. Among dentists intoxicated with occupational mercury there were significant increases in the prevalence of skin hyper-pigmentation, respiratory disorders, irregular pulse, hand tremor, spasm of upper extremities, neuropsychological symptoms, tachycardia, painful chewing, thyroid enlargement, vague fears and difficulty in writing [19]. Swedish dental workers exhibited increased central nervous symptoms [20]. Ritchie, et. al. reported increased self-reported kidney disorders and memory disturbances among dentists [21]. Female dental workers who work with mercury show an increased risk of miscarriage [22]. It has recently been demonstrated that dentists use medications for ailments that are consistent with chronic mercury poisoning at rates up to 7.5 times that of the non-dental population [23]. The issue of mercury exposure in the dental setting is not isolated to Canada or North America. Díaz Arrázola and María Armida of Columbia concluded that “*there is a total ignorance of the danger to occupational and environmental level in the use of dental amalgam and the dental professionals, education or training on the subject*” [24] (Author’s English translation of a conclusion written in Spanish). Ritchie et al. found that a significant number of dental clinics in Scotland had levels of mercury in the office air and at the inhalation point of dental staff that exceeded Occupational Exposure Standards and recommended that “greater emphasis should be made relating to safe handling of amalgam in the training and continuing professional development of dentists” [21].

The results we obtained in this study agree with some other studies of similar design, however we could find no study that simulated laboratory work of a dental student or used the exact controls that were used in this study. Pohl and Bergman found that when controls were used (water and suction and saliva evacuators) during amalgam removal, the levels at the breathing zone of the dental worker were in the range of 1-2 μg/m^3^, however when high volume suction was removed, these researchers obtained “highly fluctuating” mercury levels of mercury vapor ranging from 60 to 450 μg/m^3^[25]. Brune et al. measured both particulate and mercury vapor at the level of the breathing zone and found when water spray was removed, “*short time threshold limit values for exposure to mercury and silver were exceeded about 10 times. With water spray the mercury content was reduced to a level considerably lower than the threshold limit value, whereas the silver concentration slightly exceeded the corresponding limit*” [26]. Nimmo et al. assessed particulate levels at the inhalation area of the dentist and the patient during amalgam removal under three conditions; no water or suction(control), water and suction, and water, suction and the use of a rubber dam. The researchers found that the use of water and suction significantly reduced the respirable particulate when compared to the control and that the addition of the rubber dam reduced the particulate further, however they concluded that even with suction, water and rubber dam, the dentist was still exposed to sufficient particulate to warrant the use of a face shield during amalgam removal [27].

In our study, we assessed only dental students’ exposure to mercury vapor when amalgam is removed with a high-speed drill. The aerosol generated from drilling on amalgams also includes particulate material that is a far greater source of mercury exposure when inhaled than is mercury vapor [28]. In addition, our study did not consider the exposure to mercury via the skin, which is another known route of entry of this toxin. Other sources of mercury vapor when working with amalgam may come from amalgam placement (FDI) of the material, as well as polishing and scaling (cleaning) the surfaces of mercury amalgam fillings [29]. Future studies should be undertaken to quantify the dermal exposure to mercury of dental students as a result of exposure to both mercury vapor and mercury-containing amalgam particles. Other studies could be designed to assess the value of personal protective equipment such as mercury-resistant clothing and certified respirators [30,31] designed to reduce or prevent mercury exposure. Assessments of exposure of dental hygienists exposure to mercury vapor when polishing and scaling amalgam fillings is also highly recommended.

As the occupational risks of using amalgam continue to grow, and the availability of more effective [32-34], economical [35] and safer [36] materials such as composites exists, it appears logical that the use of amalgam be discontinued.

## Conclusions

It is paramount that dental schools consider how dental students are trained in the subject of mercury hygiene when removing dental amalgam as well as other procedures where mercury exposure may occur. They must also train dental students in the effective use of personal protective equipment in order to prevent occupational exposure to mercury while in dental school and in clinical practice. Some dental associations and government agencies have developed educational health and safety information identifying occupational hazards and control measures for dental workers. In the “Handbook of Occupational Hazards and Controls for Dental Workers” [30], Alberta Occupational Health and Safety (AOHS) summarizes the need for engineering controls and personal protective equipment when removing amalgams. In this handbook, AOHS recommends the “Elimination of mercury containing amalgams. Substitution with less harmful product(s)” as part of the major engineering control strategies to reduce the risk to dental workers. In some instances, dental schools are not presently teaching mercury hygiene to the standards set by agencies responsible for occupational safety. Dental students require mercury hygiene training so that they may integrate this knowledge into their clinical practices, thus enhancing the safety of dental staff and patients.

## Abbreviations

AOCL: Alberta occupational ceiling limit; AOTLV: Alberta occupational threshold limit value; TLV: Threshold limit value; ACGIH: American Conference of Governmental Industrial Hygienists; AOHS: Alberta Occupational Health and Safety.

## Competing interests

The authors declare that they have no competing interests.

## Authors’ contributions

BL, AO, and RW participated in the design of the study, carried out the simulations, and collected and recorded data. RW drafted the manuscript. All authors read and approved the final manuscript.
